# Bioactive Compounds of Nutraceutical Value from Fishery and Aquaculture Discards

**DOI:** 10.3390/foods10071495

**Published:** 2021-06-28

**Authors:** Mirko Mutalipassi, Roberta Esposito, Nadia Ruocco, Thomas Viel, Maria Costantini, Valerio Zupo

**Affiliations:** 1 Stazione Zoologica Anton Dohrn, Department of Marine Biotechnology, Villa Dohrn, Punta San Pietro, 80077 Naples, Italy; mirko.mutalipassi@szn.it (M.M.); thomas.viel7@gmail.com (T.V.); 2 Stazione Zoologica Anton Dohrn, Department of Marine Biotechnology, Villa Comunale, 80121 Naples, Italy; roberta.esposito@szn.it (R.E.); nadia.ruocco@szn.it (N.R.); 3 Department of Biology, University of Naples Federico II, Complesso Universitario di Monte Sant’Angelo, Via Cinthia 21, 80126 Naples, Italy

**Keywords:** wastes, seafood, aquaculture, fishery, functional foods, bioactive compounds, biotechnology, sustainability

## Abstract

Seafood by-products, produced by a range of different organisms, such as fishes, shellfishes, squids, and bivalves, are usually discarded as wastes, despite their possible use for innovative formulations of functional foods. Considering that “wastes” of industrial processing represent up to 75% of the whole organisms, the loss of profit may be coupled with the loss of ecological sustainability, due to the scarce recycling of natural resources. Fish head, viscera, skin, bones, scales, as well as exoskeletons, pens, ink, and clam shells can be considered as useful wastes, in various weight percentages, according to the considered species and taxa. Besides several protein sources, still underexploited, the most interesting applications of fisheries and aquaculture by-products are foreseen in the biotechnological field. In fact, by-products obtained from marine sources may supply bioactive molecules, such as collagen, peptides, polyunsaturated fatty acids, antioxidant compounds, and chitin, as well as catalysts in biodiesel synthesis. In addition, those sources can be processed via chemical procedures, enzymatic and fermentation technologies, and chemical modifications, to obtain compounds with antioxidant, anti-microbial, anti-cancer, anti-hypertensive, anti-diabetic, and anti-coagulant effects. Here, we review the main discards from fishery and aquaculture practices and analyse several bioactive compounds isolated from seafood by-products. In particular, we focus on the possible valorisation of seafood and their by-products, which represent a source of biomolecules, useful for the sustainable production of high-value nutraceutical compounds in our circular economy era.

## 1. Introduction

### 1.1. Fishery/Aquaculture Practices and Targeted Organisms

**Figure 1:**
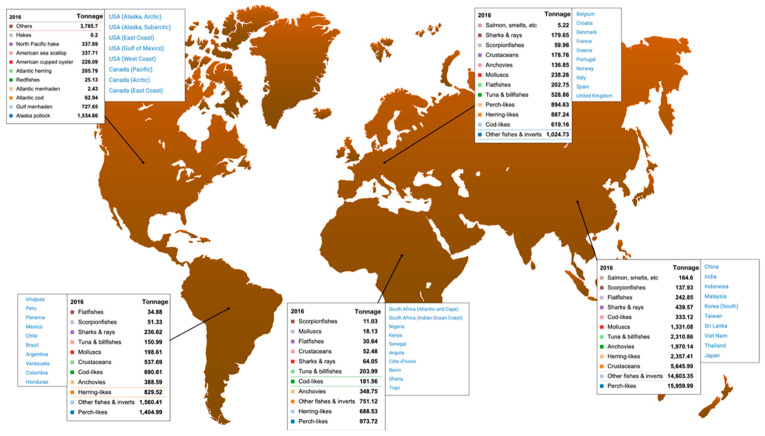
Global seafood production (updated in 2016) according to Pauly et al. [1].

Global seafood production in the year 2016 was assessed to be about 171 million tons (Figure 1) [1]. Fishery activities and aquaculture generate a wide array of different wastes. First of all, plastic wastes are heavily produced due to abandoned, lost or discarded fishing gear bilges, as well as other wastes from vessel operations. In parallel, fisheries bycatch discards are produced through low-selective fishing gears, not equipped to exclude non-targeted organisms. These latter methods may catch significant amounts of finfish species, juveniles, benthic animals, marine mammals, marine birds and vulnerable or endangered species, which are often immediately discarded. Moreover, unmarketable organisms due to small size, as well as damaged and inedible specimens, cannot be retained due to management or quota restrictions [2].

Within fisheries management, discarding is currently one of the most important issues, both from the economic and the environmental point of view [3]. The Food and Agriculture Organization (FAO) Fisheries Glossary describes it as “that proportion of the total organic material of animal origin in the catch which is thrown away or dumped at sea, for whatever reason. It does not include plant materials and postharvest wastes such as offal. The discards may be dead or alive”. In the United States, during the period 2009–2013, about 47% of the edible seafood was not used for human consumption, representing a large percentage of harvest discarded as by-catch during commercial fishing [4]. A comparable situation was found in Europe, where the ratio between seafood consumed and discarded as waste is estimated to be 1:1 [5]. Scientists and fishery managers underlined the importance of reducing these wastes, minimizing the ecological impacts of fishery [6,7,8,9,10,11,12]. In addition, a strong diversification of marine harvest was recommended, to reduce the fishing pressure on current target species by using those that are not considered interesting for commercial purposes [12].

Active bottom-contact gears (e.g., bottom trawls) are widespread large-scale fishing techniques, generally known to produce the highest discarding as compared to any other fishing gear which in many countries are becoming a real concern [2]. Thus, shrimp fisheries, particularly in tropical waters, had the highest total amount and proportion of discards with a weighted average rate of 62% [2,13,14,15]. Shrimp trawling produces the highest level of discard/catch rations if compared with other fishing techniques with values ranging from 3:1 to 15:1, according to target species, seasons and areas [16]. Along the Indian coasts, fishery bycatch is mainly composed of high proportions of juveniles and sub-adult individuals of commercially important species. It was estimated that in 2008 the annual economic loss due to juveniles in fishing, by trawlers, purse seiners, ring seiners and mini-trawlers together, was about US$ 19.445 million yr^−1^ [17]. If compared to shrimp fisheries, finfish trawling has a relatively low discard rate, contributing to a substantial total amount of discards worldwide. In addition, tuna and high migratory species contribute to total bycatch with up to 28.5% of the weighted average discard rate. In contrast, small-scale and artisanal fisheries exhibit very low or negligible discards, although in some areas, for example in the Mediterranean, the total amount of discards can still be very substantial due to a huge presence of artisanal fleets [18,19].

The taxonomic composition of discards varies among different areas and techniques according to the natural biodiversity patterns of fishing areas, target taxa, fisheries types, and gears. In the Atlantic Ocean, redfish (Sebastidae, 19%), hake (Merlucciidae, 18%), American plaice (Pleuronectidae, 13%) and rays (Rajidae, 12%) are the dominating species [2]. These organisms are potentially marketable but, when taken by fishing gears, are not retained and utilized, either because of poor product quality or small size and/or due to deliberate high-grading of catches (especially in areas outside of management intervention). In the northeast Atlantic Ocean, discarded species are quite different ranging from haddock (Gadidae, 19%), redfish (16%), Atlantic cod (Gadidae, 11%) and hake (6%). Data from eastern central Atlantic area (west Africa, FAO area 34) show a lack of resolution but it is possible to identify some taxonomically distinct entities, such as cephalopods (Cephalopoda, 4%), followed by small pelagic species (European anchovy, Engraulidae) and the European pilchard (Clupeidae). An important number of discards are “miscellaneous marine organisms”, both for pelagic and coastal fisheries, which account for up to 24% of the total discarded animals that are generally non-marketable species, such as starfish (Asteroidea, 6%) and sharks (e.g., *Prionace glauca*, Carcharhinidae) [2].

In addition to other bycatch species, jellyfish are interesting cases of study [20], since they are usually avoided by fishermen for their capability to interfere with fishing activities [21]. The occurrence of high-density gelatinous organisms during blooms are correlated with both favourable conditions in marine environments and intrinsic biological characteristics of each group [22]. In Brazil, jellyfish are identified as the organisms caught in the highest frequency and number, especially during spring and summer as in the case of the hydromedusa *Rhacostoma atlanticum*. Potential uses of jellyfish for commercial purposes are still in early stages although some studies were conducted to capture and process these organisms for human consumption. In fact, in some Asian countries, jellyfish are considered a delicacy and their commercial market is increasing [20]. In addition to this potential utilization, jellyfish are extremely rich in secondary metabolites that could find interesting applications in biotechnological fields [23]. Recently, a commercial product named Prevagen^®^, a dietary supplement containing apoaequorin, a protein extracted from the jellyfish Aequorea victoria, has been demonstrated to bind calcium in the brain, improving the electrical signals between nerve cells and contributing to prevent dementia and Alzheimer’s disease [24,25].

### 1.2. Most Useful Discards from Several Seafood Taxa

Seafood wastes are used as raw material for silage, fish meal, and fertilizer or as a component of aqua- and poultry feeds [26,27,28,29] thank to the high content of proteins, polyunsaturated fatty acids (PUFA), and other nutrients having various health benefits including carotenoids, minerals, vitamins, squalene, glycosaminoglycans. For this reason, despite the low value traditionally assigned to fishery by-products, there is a growing interest in the potential use of these wastes as functional ingredients, nutraceuticals, and pharmaceuticals in a wide range of applications [30,31,32,33,34,35,36,37]. On one side, this approach offers a significant benefit from an economical point of view, providing additional income from a material that, in some cases, has a disposal cost. On the other side, the valorization of seafood wastes, including bycatch and byproduct, can strongly reduce environmental pollution [38]. Since fishery and aquaculture wastes are rich in high-quality nutrients, there is a great potential in the marine bioprocess industry to convert and use a large fraction of these valuable products. For instance, seafood represent a rich source of proteins varying in functional and biological properties [26,39], available in high concentration in fish heads, backbones, tails etc. These proteins, as well as other biomolecules retrieved in seafood and especially in shellfish, can be easily extracted using new technologies developed for biotechnological purposes, as in the case of the chitosan produced from exoskeletons of the shrimp *Pleoticus muelleri* [40,41]. These advances include (a) macromolecules biotransformation via enzymes or microorganisms, (b) subcritical and supercritical extractions for the isolation of target products, (c) ultra-filtration, (d) microwave and (e) ultrasound-assisted recovery processes and membrane separation [37,39,42,43]. For these reasons, cheap and energy-efficient enzymatic techniques are emerging in food processing, based on the use of proteases, glycoside hydrolases, lipases, and transglutaminases [44,45].

The pre-processing operations of seafood, from fisheries and aquaculture, generate miscellaneous wastes, depending on the raw material and the desired final products in diverse markets [46,47]. Seafood processing wastes include beheading, de-shelling, skinning, gutting, removal of fins and scales, filleting, washing, etc. This waste can represent up to 40% of the total seafood. This material can be wasted as solid discards, offal, or by-product [33]. The percentage of waste materials can vary according to the processed organism, as in the case of finfish, generating up to 50% of waste material that comprises entrails, heads, skeletal frames, skin, scales, and viscera. The same wastes are produced during tuna canning operations but the process, in this case, results in a higher percentage of solid wastes (about 70%).

Crustacean wastes and byproducts can reach 75% of the shellfish, as in the case of lobster processing industries, which are composed of cephalothorax, carapace, tail, and shell [30,31,32,33,48]. Shellfish wastes are largely insoluble and very resistant to natural biodegradation, which might lead to health and environmental concerns. However, the constituents of shells, generally 30% protein and 30% chitin, make them interesting for further processing. A major problem with shrimp biomaterial valorization is the high perishability of the material that, in a tropical climate, is rapidly deteriorated by bacterial activities. Various technologies have been developed to use shrimp wastes, replace standard and hazardous chemical methods and extract bioactive compounds [49]. Shellfish are rich in carotenoids, which are lipophilic compounds responsible for yellow and red colours in nature [50] and, in particular, astaxanthin is commercially exploited due to its role as antioxidant, and in aquaculture as a feed additive for enhancing flesh colouration (i.e., the pink colour) of farmed salmonids which is generally desired by consumers [37,51,52].

In addition to fishes and crustaceans, marine algae wastes can be potentially exploited. In fact, from a nutritional point of view, edible seaweeds are rich in minerals and vitamins, being recognized as an ideal source of iodine as well as one of the few plant sources of vitamin B12 [53]. Various seaweeds have been historically harvested for human consumption and *Ulva* (Chlorophyta), *Porphyra* (Rhodophyta), *Undaria*, *Laminaria*, *Himanthalia*, and *Saccharina* (Phaeophyceae) are common ingredients of many Asian recipes [54], improving the quality of various food products [55]. Macroalgae wastes are not sufficient to satisfy worldwide demand and several algal species are intensively cultivated, especially in integrated aquacultures [56,57].

### 1.3. Aim of the Review

In the present review, we considered the studies describing the seafood-derived compounds with potential use in nutraceutical field (see Tables 1–7). Several classes of compounds were chosen, including collagen, gelatin, minerals, proteins, lipids, carotenoids, polysaccharides, and phenols. For each of them, we outlined which taxa, organism or, tissue, might be the most interesting source. When available, we highlighted the bioactivity of these compounds, demonstrated through in vitro or in vivo tests, such as antioxidant, anti-hypertensive, anti-diabetes and so on. We finally reported some examples of commercialized products containing seafood-derived compounds already used for treating human diseases. The biotechnological significance of aquaculture, fishing, and industrial by-products was thus investigated and debated, together with future expectations and challenges.

## 2. Collagen and Gelatin

Collagen type I, II, and IV have been particularly extracted from skin, bones, scales and cartilages [58], through a sustainable approach respecting the European zero-waste strategy. In fact, fish, echinoderms and jellyfish discards are suitable sources of high-quality collagen that has been efficiently extracted and processed by acid, alkaline, and enzymatic treatments coupled to mechanical methods, such as ﻿pH adjustments, homogenization, and sonication [59]. Type I collagen was extracted from the tissue of sea urchins [60,61], octopus [62], starfish [63], jellyfish [63], and several species of fish [64,65,66]. The main amino acids found within collagen and gelatin molecules are glycine (Gly), alanine (Ala), proline (Pro) and hydroxyproline, with basically a Gly-X-Y triplet as a repeating unit. Amino acids composition may differ depending on the environmental conditions (e.g., temperature), type of tissue, and extraction methods [67].

Nowadays, collagen displayed a wide range of applications in the health-related sectors, namely in cosmetics, the pharmaceutical industry and medical care (including plastic surgery, orthopaedics, ophthalmology and dentistry) [68]. Despite the high potentialities of collagen and gelatin for developing medical products and new therapeutic strategies, so far, only a few examples of commercially available drugs have been recorded [69]. Concerning non-health sectors, a noteworthy use of collagen is related to the food sector (food processing and nutraceuticals), but most often as gelatin, i.e., in its denatured form [36]. Indeed, collagen has become a functional ingredient towards the “healthy foods” development. Generally, collagen production decreases with age and bad diet [70] and consequently food supplements are intended to uphold the skin, hair, nails, and body tissues of the users [71]. Fish gelatin can be obtained by hydrolyzing and denaturing collagen [72] through a pre-treatment step, which is necessary before the extraction for improving the extraction efficiency. The pre-treatment consists of acid or alkaline hydrolysis, a method chosen according to the source material [72], and gelatin can be prepared by acid or water extraction [73]. The properties of gelatin are influenced by two main factors: the characteristics of the initial collagen and the extraction process [73,74]. Gelatin is widely used as an ingredient to improve the elasticity, texture and stability of foods but it may also give rise to biologically active peptides by protease hydrolysis. These metabolites have a potential activity as inhibitors of angiotensin I converting enzyme (ACE) or as antioxidants. For instance, gelatin obtained by acid extraction from four different species of local marine fish caught off the coast of Langkawi Island, Malaysia, such as “kerapu” (*Epinephelus sexfasciatus*), “jenahak” (*Lutjianus argentimaculatus*), “kembung” (*Rastrelliger kanagurta*), and “kerisi” (*Pristipomodes typus*) contained essential amino acids, with glycine being the most predominant (Table 1) [75]. Gelatin extracted from tunafish and giant squid tunics (*Dosidicus gigas*) demonstrated antioxidant activity after hydrolysis with trypsin, α-chymotrypsin or pepsin [76]. This work confirmed the high antioxidant capacity of whole and fractionated alcalase hydrolysates of gelatin from giant squids. This capacity was noticeably higher than that obtained from tuna fish, under the same hydrolysis conditions [76]. In a similar study, the antioxidant activities of gelatins extracted from frozen inner and outer tunics of the jumbo flying squid (*Dosidicus gigas*), tuna (*Thunnus* spp.) and halibut (*Hypoglossus* spp.) skins were evaluated by FRAP (ferric reducing antioxidant power) and ABTS (2,2′-azino-bis-3-ethylbenzothiazoline-6-sulfonic acid) assays [77]. In particular, gelatin extracted from squid showed a greater antioxidant activity due to the reduction of iron and an enhancement of the removal of free radicals [77]. Peptides purified from skin gelatin of the Pacific cod (*Gadus macrocephalus*), in particular papain hydrolysate, showed potent antioxidant activity [78]. Furthermore, the peptides obtained from the purification of papain hydrolysate showed a potential inhibitory effect of the ACE enzyme [78]. The gelatin extracted from the skin of the Chum Salmon (*Oncorhynchus keta*) and its hydrolysate had protective effects against UV irradiation-induced skin photoaging [79]. In particular, gelatin hydrolysate of the salmon skin could be used in the nutraceutical and cosmeceutical industries to reduce oxidative stress, maintain the homeostasis of the collagen matrix [80], and strengthen the immune system [81].

Ultrasound treatment for the extraction of collagen from the skin of the sea bass *Lateolabrax japonicus* has been developed by several authors [82]. In addition, collagen extracted by giant edible jellyfish, *Nemopilema nomurai*, stimulated the production of immunoglobulins and cytokines by human hybridoma cells and human peripheral blood lymphocytes without inducing allergic complications [83].

## 3. Mineral Salts

These compounds are divided into the major minerals (macro-minerals), such as calcium, sodium and potassium, and trace minerals (micro-minerals), including iron, copper, zinc, and manganese (Table 2) [84].

Among them, calcium is a fundamental element for the physiology of vertebrates, to build and maintain strong bones, as well as for many other tissues and biochemical processes [85]. Calcium extracted from food wastes showed good potentialities to make calcium-enriched bread for treating patients with osteoporosis deficiencies [86]. As demonstrated by Jung et al. [87], the skeletons discarded from industrial processing of hoki (*Johnius belengerii*) and its digested products could be used as nutraceuticals with potential calcium-binding activity. Milk tablets supplemented with nano-powdered eggshell (NPES) or nano-powdered oyster shell (NPOS), NPES with zinc (Zn-NPES), and NPOS with activated zinc (Zn-NPOS) were found suitable tools for calcium supply since did not show any significant differences in pH, consistency, colour, humidity when compared to control milk tablets [88]. Moreover, bread enriched with oyster shells showed higher protein, ash and fiber contents than control bread. In addition, the eggshell and oyster bread had significantly higher levels of calcium, iron, zinc, and phosphorus than the control. The fortification of bread with natural sources of calcium, such as skimmed milk powder and waste products, like oyster shell and eggshell powders, was demonstrated to improve the rheological characteristics of dough and the quality and nutritional properties compared to the control bread [89]. Although some studies investigated the high value of calcium from seafood by-products as food supplement, it must be considered that solubilization and ionization processes are necessary for its real adsorption. This latter issue raises not a few limits to a concrete application of marine calcium minerals for human consumption [90].

Iron is another mineral involved in many biochemical processes such as oxygen transport, energy production and cell proliferation [91] and for these reasons is one of the most important trace minerals in human physiology. However, nearly one-fifth of the population in the world reports some nutrition issues due to iron deficiency [92,93]. Therefore, iron can be administered through the diet in salts, metal chelating agents and iron-chelating peptides. For instance, the skin of the Alaska pollock hydrolyzed with trypsin generates iron-chelating peptides of high stability and adsorption features useful as iron vehicles in therapeutic food supplements [94].

Marine seaweeds contain 10–100 times more minerals than traditional vegetables with iron, copper, calcium, and magnesium present in higher concentrations [95]. Moreover, seaweeds can be considered as the best inexpensive food to fulfil the iodine requirements of humans and, more generally, they can be used as mineral food supplement [96], as well as, for their beneficial effect on hypercholesterolemia and arterial hypertension [97]. Due to their nutritional value, algae are commonly used as dietary supplements, as in the case of algae belonging to the *Spirulina* and *Chlorella* genera [55]. For example, *Laminaria japonica* is known to store marine minerals in a highly concentrated form, and it has been used to produce algae-based ingredients for skin protection against UV damage [98].

## 4. Protein and Protein Hydrolysates

Despite the availability on the market of various molecules with anti-hypertensive activity, clinical tests indicate that side effects, such as cough and angioedema, are extremely common [99]. A potential solution is to replace the synthetic inhibitors, normally used in therapeutic formulation, with natural peptides retrieved in food proteins [100]. Interestingly, marine bioactive peptides possess various biological functions, including the inhibition of ACE, antioxidant, immunomodulatory, anti-microbial, and anti-coagulant activities (Table 3) [101,102]. Fish protein hydrolysates, for their amino acid composition and easily digestible proteins, are considered to have excellent quality, from a nutritional point of view. Nevertheless, due to the unpleasant fishy smell and flavour, they were mostly used for animal nutrition [103,104]. Recent studies provided evidence that marine bioactive peptides from several marine organisms act as potential antioxidant inhibiting lipid peroxidation and removing reactive oxygen species [105,106]. Interestingly, free radicals scavenging activity has been addressed to the hydrophobic amino acids (e.g., alanine, phenylalanine, isoleucine, leucine, valine and glycine and proline, methionine, tyrosine, histidine, lysine and cysteine) that may improve the efficiency of antioxidant peptides. In fact, these amino acids could act as proton donors or electron and/or as lipid radical scavengers [107]. For instance, His-Gly-Pro-Leu-Gly-Pro-Leu (797 Da) peptides extracted from fish Hoki (*Johnius belengerii*) skin gelatin had antioxidant activity, tested in a linoleic acid peroxidation system and radical-scavenging potency. In addition, antioxidative enzyme levels in cultured human hepatoma cells increased in the presence of this peptide, suggesting that it was involved in maintaining the redox balance in the cell environment [108]. Alkalin-pretreated cobia (*Rachycentron canadum*) skin was extracted in a retort for 30 min to obtain a retorted skin gelatin hydrolysate (RSGH). Cobia RSGH and its derivatives showed a strong antioxidant activity by inhibiting lipid peroxidation. It is well-known that lipid peroxidation occurring in food products deteriorates food quality, resulting in rancidity, unacceptable taste, and shorter shelf-life. The RSGH retarded lipid deterioration and may be used as a natural antioxidant for food products. In fact, these peptides can be used as antioxidants in functional foods and supplements [109]. In another study, antioxidant activity of fish protein hydrolysates obtained from cod backbones (*Gadus morhua*) was evaluated using liposomes and DPPH (2,2-diphenyl-1-picrylhydrazyl) radical scavenging assay. Moreover, the DPPH scavenging activity showed that the anti-oxidative activity of hydrolysates could be due to the ability to scavenge lipid radicals [110]. The protein hydrolysates obtained through different enzymatic treatments from sardine heads and/or entrails (*Sardinella aurita*) [111,112], Alaska pollack (*Theragra chalcogramma*) skin [113], and from the muscles of the sea bream (*Nemipterus japonicus*) and other fish (*Exocoetus volitans*) showed antioxidant activity. Moreover, the trypsin protein hydrolysates of both fishes showed maximum free radical scavenging potential and lipid peroxidation inhibition. Furthermore, these peptides showed a significant anti-proliferative effect on Hep G2 (human hepatocellular liver carcinoma) cell line [114]. A peptide was isolated from the Black pomfret (*Parastromateus niger*) viscera, showing a peculiar antioxidant activity, able to inhibit lipid peroxidation and oxidative damage [115]. A similar activity was also identified in Horse mackerel visceral protein hydrolysate from the fish *Magalaspis cordyla*. In particular, this peptide inhibited lipid peroxidation avoiding oxidative damage in living systems [116]. In vitro assays reported antioxidant activities in two peptides isolated from skin protein hydrolysates of the horse mackerel (*Magalaspis cordyla*) and the croaker (*Otolithes ruber*) [117,118], and eight hydrolysates from cuttlefish (*Sepia officinalis*) by-products (skin and viscera) obtained through treatment with various gastrointestinal proteases (chymotrypsin, trypsin, and crude alkaline enzyme and bacterial proteases) [119]. As reported for the antioxidant properties, ACE inhibitory activity was also attributed to the differences in chain length and amino acids sequences of peptides, as well as, to their hydrophobicity [120]. For instance, an ACE inhibitory Gly-Leu-Pro-Leu-Asn-Leu-Pro (M.W. 770 Da) hydrophobic peptide isolated from salmon skin (*Oncorhynchus keta*) was found to reduce systolic blood pressure after oral administration in rats, suggesting a possible use of this peptide as a functional food with anti-hypertensive effect [121]. Moreover, the jellyfish aqueous/hydroalcoholic extracts and the hydrolyzed peptides resulting from pepsin and collagenase digestions obtained from three Mediterranean species of jellyfish (*Aurelia* sp., *Cotylorhiza tuberculate*, and *Rhizostoma pulmo*) showed evident antioxidant activity, with suitable application in nutraceutical, cosmeceutical, and pharmacological fields [122]. Antioxidant and ACE inhibitory molecules can be found in other jellyfishes, as in the case of *Rhopilema esculentus* Kishinouye, where a Ser-Tyr dipeptide abundant in the gonads of this species, had DPPH, hydroxyl and superoxide radical scavenging effects with IC_50_ 84.623 µM, 1177.632 µM, 456.663 µM, respectively [123,124].

As mentioned before, macroalgae can be also used for the development of nutraceutical products. Particularly interesting is the case of two peptides (Gly-Gly-Ser-Lys and Glu-Leu-Ser) identified from proteolytic enzymes hydrolysates isolated from the red seaweed laver (*Porphyra* species), that significantly inhibited α-amylase activity at 1 mg/mL by colorimetric method. Since this enzymatic activity can reduce blood glucose levels, the potential application of seaweed hydrolysates in diabetes treatments has been proposed [125]. Similarly, a novel peptide isolated from Nori hydrolysate inhibited the clotting factors involved in the intrinsic pathway of coagulation and, for this reason, it could be used as a functional food in the prevention of thrombosis [126].

## 5. Lipids

Lipids belong to a fundamental group of nutrients for humankind since they contribute the structure of the biological membranes [127], and act both as energy storage and key signalling molecules [128]. Their components are fatty acids (FAs), which could be classified into saturated (SFAs—without double bonds), monosaturated (MUFAs—with one double bond), and PUFAs (with two or up to six double bonds) [129]. Nowadays, essential FAs are considered to be functional foods and nutraceuticals with many benefits for human health, including the potential of reducing the risk of cardiovascular diseases, cancer, osteoporosis, diabetes [129], inflammation and neurocognitive function [130], and autoimmune diseases [131].

Among lipids, lecithin is a sticky fatty substance mainly composed of phospholipid mixtures with a small percentage of glycerides, neutral lipids, and other suspended matter. Lecithin was used for its emulsifying properties in nutraceutical (i.e., lecithin nanovesicles as supplementary food) [132], pharmaceutical (i.e., hypercholesterolemia, neurologic disorders and liver ailments) [133], and cosmetic sectors (i.e., beauty lotions and cosmetic oil) (Table 4) [134]. Marine lecithin was mostly isolated and characterized from squid (*Todarodes pacificus*) viscera residues de-oiled by supercritical carbon dioxide (SC-CO_2_) extraction. In particular, the main phospholipids of lecithin from squid viscera were phosphatidylcholine and phosphatidylethanolamine [135,136]. Some differences in the composition of the phospholipids of squid viscera arose [137] and can be probably explained by variations in habitat, food intake, fishing seasons of sampled organisms, and isolation/quantification processes. However, in other studies, the composition of these phospholipids from squid viscera was almost the opposite [137].

Lipids extracted from the liver of the Australian lobster (*Jasus edwardsii*), using SC-CO_2_, contained high concentrations of PUFA and low levels of contaminants such as lead, arsenic, mercury and cadmium [138]. So, lipids extracted from the liver of lobsters may be useful in the prevention and treatment of several disorders and diseases including coronary heart disease, rheumatoid arthritis, asthma, cancers, diabetes. A deep red oil rich in omega-3 PUFAs, especially EPA and DHA, can be also obtained from the by-products (head, shell and tail) of the Northern shrimp (*Pandalus borealis*) [139] and from the ground skin of the Indian mackerel (*Rastrelliger kanagurta*). In particular, oil extracted from Indian mackerel had the highest recoveries of PUFAs [140]. When ethanol was added as a co-solvent of SC-CO_2_, higher recovery of PUFAs, especially DHA, omega-3, and omega-6, from fish by-products (e.g., head of the longtail tuna *Thunnus tonggol*) was obtained [141]. Interestingly, oils extracted from various salmon by-products (belly part, trimmed muscle, frame bone and skin) with different techniques (hexane extraction, SC-CO_2_ and pressed oil) did not show any differences in fatty acid composition. However, significant variation was detected in free radical scavenging activity, since oils extracted by SC-CO_2_ exhibited greater antioxidant properties than those extracted by hexane [142].

A good example of commercialised product is Lyprinol^®^, a lipid fraction of the freeze-dried extract from the farmed green-lipped mussel *Perna canaliculus*. Due to its anti-inflammatory activities, the drug is currently sold to reduce the inflammatory processes related to arthritis [143,144].

Considering lipids from waste sources, it is important to underline the importance of squalene as a bioactive molecule. Squalene is a natural lipid belonging to the terpenoid family, which partly originates from endogenous cholesterol synthesis and partly from dietary sources, especially in populations consuming large amounts of olive oil or shark liver, olives, wheat germ, and rice bran [145]. Squalene is considered an excellent emollient and moisturizer for the skin, also having antioxidant and anti-cancer properties, to relieve skin irritations and/or tumors [146,147].

A few words should also be addressed to vitamin E, which is a lipid-soluble antioxidant occurring both in plants and animals for the protection of biological membranes against lipid peroxidation. Four homologue pairs (α-, β-, γ-, δ-tocopherols and -tocotrienols) have been described, with the α form being the most active [148]. Marine tocopherols have been firstly isolated from salmon eggs and subsequently from tissues and organs of several fishes, making them a considerable source of these beneficial molecules [149].

## 6. Carotenoids

Salmons, crustaceans, and shrimps processing wastes could be an important source of carotenoids (e.g., astaxanthin; Table 5) [150,151,152]. On one side, the intake of carotenoids from food or supplementation should be carefully considered due to the lack of specific biosynthetic pathways in humans [153]. On the other side, carotenoids are interesting bioactive molecules able to mitigate the damaging effect of oxidative stress [154]. In particular, a positive link between higher dietary intake, tissue concentrations of carotenoids, and lower risk of chronic diseases was found [155]. Moreover, the antioxidant activity of astaxanthin modulates the biological functions related to lipid peroxidation, having beneficial effects on chronic diseases, such as cardiovascular diseases, macular degeneration, and cancer [150]. In the specific case of astaxanthin, [156] its oral administration of 1 mg/kg/day for 14 days significantly reduced hepatic metastasis in rats, suggesting an important role in enhancing the immunological response through the inhibition of stress-induced lipid peroxidation. Astaxanthin and its esters displayed a strong antioxidant activity with increasing extract concentration [157]. Furthermore, astaxanthin had an anti-proliferative effect on human laryngeal carcinoma cells (Hep 2 cells), as demonstrated by Sila et al. [157]. Interestingly, to improve the stability of astaxanthin obtained from shrimp shells (*Litopenaeus vannamei*), encapsulation in alginate-chitosan beads was attempted for allowing its real use as a functional ingredient [158].

The content of fucoxanthin, a carotenoid extracted from the macroalga *Undaria pinnatifida*, is also used to control eutrophication and aids the sustainable development of aquaculture [159]. It was lower than the fucoxanthin content found in Japanese commercial wakame products, probably because of the different places and months of collection. Fucoxanthin extracted from fresh samples exerted a potent antioxidant activity that was higher than the commercial algae due to the loss of phenols and fucoxanthin during processing [160]. Similarly, fucoxanthin extracted from the species *Sargassum wightii* Greville showed antioxidant activity in vitro and inhibition of ACE, with potential application as a food ingredient to overcome hypertension [161].

## 7. Polysaccharides

Crustaceans, shrimps, and crabs are the main sources of chitin from the sea [162]. The most important derivative of chitin is chitosan, obtained through partial deacetylation of chitin under alkaline conditions or enzymatic hydrolysis in the presence of a chitin deacetylase [162]. Several studies proved that chitosan and its derivatives have antioxidant, anti-microbial and anti-viral activities [163,164,165,166]. Chitin, chitosan, and their derivatives also act as inhibitors of ACE, an enzyme associated with hypertension. Chemical methods for obtaining chitin from marine wastes (shrimp shells and crabs) provided demineralization and deproteinization, with the use of strong acids or base. Meanwhile, biological methods utilize enzymatic treatment by protease and microbial fermentation [167].

Interesting studies demonstrated the availability of chitin and chitosan in various marine organisms, as in the case of the eggs of the snail *Rapana venosa* and the exoskeleton of the marine crab *Eriphia verrucosa* [168] or the scales of the Red snapper fish (*Lutjanus* sp.) [169], containing chitosan available for biotechnological, agricultural and industrial purposes (Table 6). Moreover, chiton shells were also found to contain a higher abundance of chitin and chitosan than commercial products [170], and *Sepia prashadi* cuttlebone, which proved to store a great content of these compounds, as compared to other sources (e.g., crab shells) [171]. Sulfated polysaccharides extracted from the macroalgae *Gracilaria caudata* and *Gracilaria debilis* by enzymatic and water extraction, respectively, showed antioxidant activity in a concentration-dependent manner [172,173]. The antioxidant capability of these polysaccharides was evaluated in vitro by ferrous ion chelating ability and total antioxidant capacity and in vivo using an oxidative stress rat model induced by 2,2′-azobis (2-methylpropionamidine) dihydrochloride (ABAP) [173].

Carbohydrate complexes, named glycosaminoglycans (GAGs, e.g., chondroitin sulphate, dermatan sulphate, hyaluronic acid), are another class of polysaccharides with interesting bioactivities including, anti-viral, anti-metastatic, anti-inflammatory and anti-coagulant ones, plus a great potentiality in tissue engineering. The therapeutic properties normally depend on the amount and pattern of sulfate groups along the disaccharide chain. GAGs have been extracted from numerous marine organisms that may represent a seafood waste of fishery, aquaculture and industrial processes such as, sea urchins, sea cucumbers, sea squirts and shrimps [174,175,176,177]. GAGs extracted from the mussel *P. canaliculus*, were found to exert an important role as anti-arthritic agents. Studies were conducted on the commercial product Biolane™, which contains GAGs plus matrix metalloprotease (MMP’s), a family of enzymes necessary for normal tissue re-modelling. The main results revealed that this marine-derived mixture exhibited a wide range of beneficial activities, such as inhibition of pro-inflammatory prostaglandins (PGEs), cyclooxygenase-2 (COX-2), together with anti-platelet aggregation, and fibrinolytic potencies [178]. The cold extract from the same mussel species, extremely rich in glycosaminoglycans, was clinically proven to reduce joint pain and enhance joint mobility. It was commercialized as GlycOmega-PLUS™ [179].

Alginate is a polysaccharide found in the intercellular matrix of brown algae extremely rich in sodium, calcium, magnesium, strontium, and barium ions. Alginate is widely used in industry for its ability to retain water and for its gelling, viscosifier and stabilizing properties [180]. However, in addition to its use in the textile industry as a printing paste, alginate could find several applications in nutraceutical field. Alginate extracted through different techniques (water, acid, alkalase, and cellulase) from the alga *Sargassum angustifolium* showed antioxidant activity in a dose-dependent manner [181]. The brown seaweed cell wall and some marine invertebrates contain also a group of fucose-rich sulfated heteropolysaccharide compound, named fucoidan, which was consumed as dietary fiber in many Asian countries [182]. The structure of the fucoidan varies among species, but usually it contains L-fucose and sulfate, along with small quantities of D-galactose, D-mannose, D-xylose, and uronic acid. These fucoidans revealed various significant biological activities, such as antioxidant, anti-inflammatory, anti-allergic, anti-tumour, anti-obesity, anti-coagulant, anti-viral, anti-hepatopathy, anti-uropathy, and anti-renalpathy effects [183]. For instance, fucoidan extracted from the brown alga *Sargassum polycystum* showed antioxidant activity at 1000 µg/mL and anti-proliferative activity with IC_50_ of 50 µg/mL against the human breast cancer cell line [184]. In addition, fucoidan isolated from *Sargassum wightii* was found to regulate postprandial hyperglycemia in diabetic patients acting as α-D-glucosidase inhibitor in a dose-dependent manner [185].

## 8. Phenols

As part of both animal and human diet, the nutraceutical properties assigned to phenolic compounds are almost endless including protective effects against cardiovascular disease, neurodegeneration, and cancer [186]. Macroalgae phenolic compounds, particularly phlorotannins, gained particular attention due to their specific bioactivities, including antioxidant, anti-proliferative, or anti-diabetic, despite the high abundance of polysaccharides on the macroalgae matrix made the isolation and characterization quite difficult [186]. Few examples of phenolic bioactive compounds have been reported in the literature (Table 7). For instance, a 2,5-dihydroxybenzoic acid isolated from the macroalgae *Laminaria digitata* and *Undaria pinnatifida* displayed a potent α-amylase inhibitory activity [187], whereas the polyphenol-rich extract isolated from the seaweed *Sargassum vachellianum* showed a good free radical scavenging ability, anti-microbial activity and effectively absorbed the UVB and UVA rays [188]. In contrast, another marine polyphenol (Dieckol), isolated from the brown alga *Ecklonia cava*, showed sleep-enhancing effects by increasing the amount of non-rapid eye movement sleep and decreasing wakefulness during the same hours. These results implied that Dieckol can be used as a promising herbal sleep aid with minimal side effects, unlike the existing hypnotics [189]. These macroalgae represent a considerable source of waste products since they are used in intensive aquaculture and management of the eutrophication phenomenon [159,190].

## 9. Industrial Status and Trends

In the last ten years, 620 scientific contributions related to marine biotechnology have been published, with a particular focus on the pharmacological and food industry [191]. More than 1000 novel compounds have been, annually, described with a focus on seafood wastes, and several examples of nutraceutical products are already sold in the market [192]. Several companies, such as Aquapreneur (www.aquapreneur.com, accessed date 15 March 2021), Sederma (http://www.sederma.fr, accessed date 15 March 2021), NutraIngredients (www.Nutraingredients.com, accessed date 15 March 2021), SpecialChem (http://www.specialchem4cosmetics.com, accessed date 5 April 2021), Fortitech (fortitechpremixes.com, accessed date 5 April 2021), Copalis (http://www.copalis.fr/, accessed date 5 April 2021), and so on, are currently working on new marine ingredients made of collagen, various peptides, GAGs, oils, calcium supplements, and classes of compounds described above (see Section 2, Section 3, Section 4, Section 5, Section 6, Section 7 and Section 8).

Food supplements containing tripeptides, dipeptides, and also free amino acids from fish gelatin and collagen are already available commercially for the preservation of bones and tendon integrity [193]. Moreover, a huge production of fish oil as nutraceutical products was also performed all over the world. Oils contain elevated levels of long-chain omega-3 PUFAs, exhibiting beneficial activities [194]. For instance, Lovaza^®^, which contains ethyl esters of EPA (20:5) and DHA (22:6), is now available in the market to treat diabetes and cardiovascular diseases (https://www.drugs.com/pro/lovaza.html, accessed date 15 March 2021). Concerning chitosan and its derivatives, at present, several companies are involved in the production of medical and food products with nutraceutical purposes. Common examples are Seatone^®^ and Lyprinol^®^ obtained from mussels that are now sold as functional foods in anti-arthritic and anti-inflammatory treatments [195]. Concluding, it must be considered that additional compounds are still the subject of clinical trials, as in the case of the fish hydrolysate Gabolysat [196], and the mussel and fish neurotoxin Tetrodotoxin (TTX), which is investigated for its analgesic properties [197].

## 10. Conclusions and Future Perspectives

This review is aimed at an environmentally friendly and sustainable use of marine resources, to foresee possible economic benefits for the sector. The data reported show that seafood by-products contain a range of valuable biomolecules, fully appreciating what is usually considered a “waste” and exploiting them to improve human wellness and health. In ancient times, hunting, fishing and gathering were three fundamental practices for food supply. Nowadays, humans still relieve on marine natural resources as one of the main ingredients for human consumption. Since oceans occupy more than 70% of the Earth’s surface, their high biodiversity makes them a target for searching raw resources, including natural and bioactive compounds. With the increase of the global human population, marine organisms play a role not only as a supply of high-quality food, but also as a source of various compounds for pharmaceuticals, cosmetics, and nutraceutical industries. In fact, several bioactive molecules were isolated from the sea, with beneficial properties including antioxidant, anti-microbial, anti-diabetic, anti-proliferative, anti-obesity, anti-Alzheimer, anti-fibrotic, neuroprotection, sleep-enhancing, lipid-lowering, wound healing, and skin protection activities. Exploiting marine resources in a sustainable way, to satisfy the food requirement of the growing human population, puts high pressure on the natural resources of the planet. Hence, more rational exploitation of the available natural assets should be adopted. It is imperative to develop functional foods from marine products, and in particular from fishery discards, since they are widely available and they can prevent or cure various diseases. Ineffective use of the marine raw materials and the common use of non-selective fishing gears generate a loss of up to 50% of the marine captures, that are discarded in the sea, and up to 80% of the seafood raw material, that is not processed and discarded. A huge amount of wastes generated during seafood industrial processing can be properly handled to obtain raw materials. This management requires a green revolution in industrial processing with the integration of standard processing methods with environmentally friendly and cost-effective ones, to achieve sustainable production with a low ecological footprint. In addition, seafood discards are considered hazardous to the environment and can create a serious waste disposal problem. Here, we reported data demonstrating the opportunity to effectively reuse wastes and by-products from aquaculture and fisheries, which would potentially go to waste, rather than being used to produce high-quality nutraceuticals for human consumption.

The identification of functional ingredients and their nutraceutical application is a growing field. Interesting compounds, such as bioactive peptides, polysaccharides, polyunsaturated fats, carotenoids, polyphenolic compounds, minerals, collagen, gelatin, saponins, phycobiliproteins, and phytosterols, are in fact abundant in fish bycatch and food industrial scraps. The major advantages of extracting waste-derived nutraceuticals, besides their low costs, are found in the easy availability of raw materials, high recovery rates, interesting functional properties of the isolated substances, and the aforementioned potential applications.

In addition, the use of waste compounds opens new perspectives in integrative aquaculture. For example, with the potential use of edible seaweeds in phyto-depuration techniques applicable for multispecies culture systems, species can be sold as food or food complements after the production cycle. Indeed, the use of wastes in nutraceuticals has led to various answers to common issues, such as recycling, increasing of profits by industries, reduction of human footprint activities, and sustainability of marine sources.

However, many aspects should be faced, as in the case of the relationships between processing procedure and the functionality of final products. Further studies are required to evaluate the best procedures to assure the stability of marine bioactive molecules during the processing and storage, as well as the uniformity of the bioactive contents according to natural variability over the fishing areas, the seasons, and the production processes themselves. These latter issues are still limiting the successful exploitation of seafood by-products for the food industry, and further research is needed to bypass them and allow the effective production of compounds for human wellbeing.

## Figures and Tables

**Table 1:** Gelatin and collagen from seafood wastes, together with taxa, species and tissue from which they were isolated, and their biotechnological application. N/A refers to “not applicable”.

Taxa	Species	Tissue	Product	Biological Activity	Reference
Fish	*Epinephelus sexfasciatu*, *Lutjianus argentimaculatus*, *Rastrelliger kanagurta*, *Pristipomodes typus*	N/A	Gelatin	N/A	[75]
Bivalve	*Dosidicus gigas*	Tunics	Gelatin	Antioxidant	[76]
Bivalve and fish	*Dosidicus giga*, *Thunnus* spp., *Hypoglossus* spp.	Inner and outer tunics	Gelatin	Antioxidant	[77]
Fish	*Gadus macrocephalus*	Skin	Gelatin	Antioxidant	[78]
Fish	*Lateolabrax japonicus*	Skin	Collagen	N/A	[82]
Fish	*Oncorhynchus keta*	Skin	Gelatin	Antioxidant	[79]
Jellyfish	*Nemopilema nomurai*	Body	Collagen	Antioxidant	[83]

**Table 2:** Minerals and peptides carrying minerals from seafood wastes, together with taxa, species and tissue from which they were isolated, and their biotechnological application.

Taxa	Species	Tissue	Product	Biological Activity/ Nutraceutical Application	Reference
Fish	*Johnius belengerii*, *Thunnus thynnus*	Skeletons	Phosphopeptide	Potential calcium-binding activity/food supply	[87]
Bivalve	N/A	Shell	Calcium	Food supplement	[88]
Bivalve	N/A	Shell	Calcium, iron, zinc and phosphorus	Food supplement	[89]
Fish	*Gadus chalcogrammus*	Skin	Peptides	Iron-chelating/food supply	[94]
Brown alga	*Laminaria japonica*	NA	Minerals	Antioxidant	[98]

**Table 3:** Proteins and protein hydrolysates from seafood wastes, together with the taxa, species and tissue from which they were isolated, and their biotechnological application.

Taxa	Species	Tissue	Product	Biological Activity	Reference
Fish	*Johnius belengeri*	Skin	Peptides	Antioxidant	[108]
Fish	*Rachycentron canadum*	Skin	Gelatin derivate	Antioxidant	[109]
Fish	*Gadus morhua*	Backbones	Protein hydrolysates	Antioxidant	[110]
Fish	*Sardinella aurita*	Heads and/or entrails	Protein hydrolysates	Antioxidant	[111,112]
Fish	*Theragra chalcogramma*	Skin	Peptides	Antioxidant	[113]
Fish	*Nemipterus japonicus*	Muscles	Hydrolysates and peptide fractions	Antioxidant	[114]
Fish	*Exocoetus volitans*	Muscles	Hydrolysates and peptide fractions	Antioxidant and anti-tumor	[114]
Fish	*Parastromateus niger*	Viscera	Peptides	Antioxidant	[115]
Fish	*Magalaspis cordyla*	Viscera	Peptides	Antioxidant	[116]
Fish	*Magalaspis cordyla*	Skin	Peptides	Antioxidant	[117]
Fish	*Otolithes ruber*	Skin	Peptides	Antioxidant	[118]
Bivalve	*Sepia officinalis*	Skin and viscera	Protein hydrolysates	Antioxidant	[119]
Fish	*Oncorhynchus keta*	Skin	Peptides	Anti-hypertensive	[121]
Jellyfish	*Aurelia* sp., *Cotylorhiza tuberculate*, *Rhizostoma pulmo*	Body	Hydrolyzed peptides	Antioxidant	[122]
Jellyfish	*Rhopilema esculentus*, *Kishinouye*	Gonads	Protein hydrolysates	Antioxidant	[123,124]
Red alga	*Porphyra* spp.	Leaf	Peptides	Anti-diabetic	[125]
Red alga	*Porphyra yezoensis*	Leaf	Peptides	Anti-thrombotic	[126]

**Table 4:** Lipids from seafood wastes, together with taxa, species and tissue from which they were isolated, and their biotechnological application.

Taxa	Species	Tissue	Product	Biological Activity	Reference
Cephalopod	*Todarodes pacificus*	Viscera residues	Lecithin	Emulsifying properties	[135,136]
Crustacean	*Jasus edwardsii*	Liver	PUFA	Anti-inflammatory, anti-hypertensive, anti-diabetic	[138]
Crustacean	*Pandalus borealis*	Head, shell and tail	EPA and DHA	Anti-inflammatory, anti-hypertensive, anti-diabetic	[139]
Fish	*Rastrelliger* *kanagurta*	Ground skin	EPA and DHA	Anti-inflammatory, anti-hypertensive, anti-diabetic	[140]
Fish	*Thunnus tonggol*	Head	DHA, omega-3 and -6 FAs	Anti-inflammatory, anti-hypertensive, anti-diabetic	[141]
Fish	*Salmo salar*	Belly part, trimmed muscle, frame bone and skin	Oil	Free radical scavenging	[142]
Bivalve	*Perna canaliculus*	Body	PUFA/Lyprinol^®^	Anti-inflammatory and anti-arthritis	[143,144]

**Table 5:** Carotenoids from seafood wastes, together with taxa, species and tissue from which they were isolated, and their biotechnological application.

Taxa	Species	Tissue	Product	Biological Activity	Reference
Crustacean	*Litopenaeus vannamei*	Shell	Astaxanthin	Antioxidant	[158]
Brown alga	*Undaria pinnatifida*	Leaf	Fucoxanthin	Antioxidant	[159]
Brown alga	*Sargassum wightii*	Leaf	Fucoxanthin	Antioxidant and ACE inhibition	[161]

**Table 6:** Polysaccharides from seafood waste together taxa, species and tissue from which they were isolated, and their biotechnological application.

Taxa	Species	Tissue	Product	Biological Activity	Reference
Mollusc	*Rapana venosa*	Eggs	Chitin and chitosan	Antioxidant, anti-microbial, anti-viral and anti-hypertension	[168]
Crustacean	*Eriphia* *verrucosa*	Exoskeleton	Chitin and chitosan	Antioxidant, anti-microbial, anti-viral and anti-hypertension	[168]
Fish	*Lutjanus* sp.	Scales	Chitin and chitosan	Antioxidant, anti-microbial, anti-viral and anti-hypertension	[169]
Mollusc	Several species of chiton	Shell	Chitin and chitosan	Antioxidant, anti-microbial, anti-viral and anti-hypertension	[170]
Cephalopod	*Sepia prashadi*	Cuttlebone	Chitin and chitosan	Antioxidant, anti-microbial, anti-viral and anti-hypertension	[171]
Red alga	*Gracilaria caudata*, *Gracilaria* *debilis*	Leaf	Sulfated polysaccharides	Antioxidant	[172,173]
Bivalve	*Perna* *canaliculus*	Body	Glycosaminoglycans/ Biolane™	Anti-inflammatory	[178]
Bivalve	*Perna* *canaliculus*	Body	Glycosaminoglycans/ GlycOmega-PLUS™	Anti-arthritic	[179]
Brown alga	*Sargassum* *angustifolium*	Leaf	Alginate	Antioxidant	[181]
Brown alga	*Sargassum* *angustifolium*	Leaf	Fucoidan	Antioxidant, anti-inflammatory, anti-allergic, anti-tumor, anti-obesity, anti-viral	[183]
Brown alga	*Sargassum* *polycystum*	Leaf	Fucoidan	Antioxidant	[184]
Brown alga	*Sargassum wightii*	Leaf	Fucoidan	Anti-diabetic	[185]

**Table 7:** Phenols from seafood wastes, together with taxa, species and tissue from which they were isolated, and their biotechnological application.

Taxa	Species	Tissue	Product	Biological Activity	Reference
Brown alga	*Laminaria digitata, Undaria pinnatifida*	Leaf	2,5-dihydroxybenzoic acid	Anti-diabetic (α-amylase inhibition)	[187]
Brown alga	*Sargassum vachellianum*	Leaf	Polyphenol-rich extract	Free radical scavenging, antimicrobial activity and anti-UV	[188]
Brown alga	*Ecklonia cava*	Leaf	Dieckol	Sleep-enhancing	[189]
